# The Osteometry of Equine Third Phalanx by the Use of Three-Dimensional Scanning: New Measurement Possibilities

**DOI:** 10.1155/2017/1378947

**Published:** 2017-01-11

**Authors:** Sławomir Paśko, Małgorzata Dzierzęcka, Halina Purzyc, Anna Charuta, Karolina Barszcz, Bartłomiej Jan Bartyzel, Marcin Komosa

**Affiliations:** ^1^Faculty of Mechatronics, Virtual Reality Technologies Department, The Institute of Micromechanics and Photonics, Warsaw University of Technology, Warsaw, Poland; ^2^Faculty of Veterinary Medicine, Department of Morphological Science, Warsaw University of Life Sciences, Warsaw, Poland; ^3^Faculty of Veterinary Medicine, Department of Animal Physiology and Biostructure, Wrocław University of Environmental and Life Sciences, Wrocław, Poland; ^4^Vertebrates Morphology Department, Siedlce University of Natural Sciences and Humanities, Siedlce, Poland; ^5^Department of Animal Anatomy, Institute of Zoology, Poznań University of Life Sciences, Poznań, Poland

## Abstract

This study consisted in analyzing the asymmetry between bilateral third phalanges (coffin bones) in cold-blood horses based on the angle range of the plantar margin of the bone. The study employed a scanner projecting a hybrid set of images, consisting of sinusoidal stripes preceded by a Gray code sequence. As it turned out, three-dimensional scanning can be used to effectively determine the angle range for a selected portion of the studied bone. This provides broad possibilities for osteometric studies, as it enables the determination of angle distribution in a given fragment. The results obtained indicate a weak correlation between age and bilateral third-phalanx asymmetry in terms of the angle range of the plantar margins and no correlation between body weight and the asymmetry described.

## 1. Introduction

Structured-light scanning of objects is increasingly used in medicine and related areas of research in the world [1–3]. Most commercially available scanners are unidirectional, allowing the measurement of an object's shape or curvature from one direction only. These include devices using the moiré effect. Devices of this type were the first available ones, applied in research as early as the 1970s. The pioneer of such studies and the first author to capture and publish the image of a patient's back with moiré patterns projected was Takasaki [4]. More advanced devices, allowing for more precise measurement, are structured-light optical scanners [5]. These can be divided into five categories, depending on the pattern type and projection method. The scanner described by Posdamer and Altschuler [5] belongs to the category using a sequential projection of several rasters. These can be Gray codes, phase-shifted sinusoidal stripes, or binary codes; a hybrid method may also be used, applying both Gray codes and sine stripes. The hybrid method was proposed in 1998 by Brenner et al. [6]. Another group includes devices whose operation is based on color light projection. Two subgroups can be identified: one comprises devices termed “Rainbow 3D Cameras” [7] and the other comprises devices projecting continually changing color stripes [8]. Another group of scanners, rarely used commercially, operate based on the indexing of stripes [9] or parts of the projected grid [10]. It is also possible to combine selected methods from two or more categories to develop hybrid scanning methods.

The present study employed a scanner projecting a hybrid set of images, consisting of sinusoidal stripes preceded by a Gray code sequence [11]. The scanning device may also be portable, which is especially important in measurements of large animals. Another advantage of this particular method is that it allows for contactless measurement, which significantly facilitates the measurement of animals, especially agricultural ones. Furthermore, the method provides a complete set of data in a significantly reduced time. The technique enables the creation of three-dimensional images of anatomical structures. In many cases, it may also complement or even replace the traditional measurement and recording methods used in osteometry [12–19].

With regard to problems necessitating the use of bone measurement techniques, an especially interesting one is bilateral skeletal asymmetry. It has been described as common both in humans and in animals [20–22]. Marked asymmetry may result in musculoskeletal system disorders, which is particularly significant in the case of animals kept for recreation or sports purposes, including horses. A considerable number of injuries occur in the distal portions of thoracic limbs; therefore, the present study focused on the distal third phalanx, also called the coffin bone. This bone bears the entire weight of the horse's body due to the vertical position of the equine autopodium. Moreover, the distal phalanx structure in the species is incomparable with other bone structures. Many bones, including the coffin bones studied here, are complex structures, very difficult to assess with objective measurement [23]. Therefore, structured-light scanning of the bone may be a potential method of obtaining information on its shape and structure. Notably, this method had never been used for osteometric measurements before. Thus, the present study is a pioneering one.

## 2. Materials and Methods

The study material comprised bilateral thoracic limb coffin bones of the Polish cold-blood horse, mainly bred and kept as a draft horse [18]. The animals (*n* = 38) were sold from private farms to the Rawicz horse slaughterhouse for reasons unrelated to any musculoskeletal disorders. Bones for analysis were isolated from limbs of randomly selected horses. According to the Polish law, the postmortem use of tissues does not require an approval from the Ethics Committee [24].

**Figure 1:**
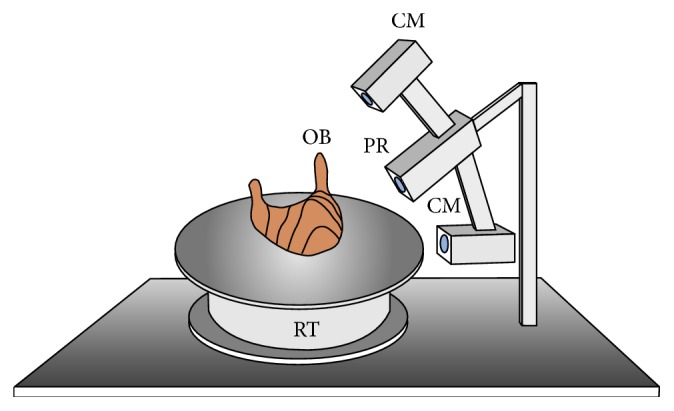
Diagram of the scanning system used for scanning the bones. CM: camera; PR: projector; RT: rotary table; OB: object.

At the first stage of the study, a part of the autopodium comprising the hoof was separated by severing the pastern joint. Subsequently, each hoof was marked. Then, it was bagged and immersed in water at 95–99°C for 48 h, after which the coffin bone was isolated from the hoof capsule, cleaned, and air-dried at room temperature for a week. Each coffin bone was marked on the articular surface with the number of the horse and a letter indicating the left or right limb. The isolated, dried, and marked coffin bones were laid on the flat surface of the rotary table comprised in the measurement system (Figure 1). The surface was level and covered with antislip fabric. Therefore, no fastening of the studied bones to the surface was necessary to prevent displacement during the movement of the table.

The other part of the measurement system was the 3D scanner installed at the appropriate distance (Figure 1). The scanner operated as follows: the measured object (OB), that is, the coffin bone, was placed on the rotary table (RT), and a stripe pattern was projected upon it from one direction by the projector (PR). The patterns were observed using two cameras, one above and one below the projector. The apparent distortion of the patterns provided information on the shape of the object [11]. The distance between the cameras was 500 mm, and the distance between each camera and the projector was 250 mm. The first camera was positioned at a 60° angle to the projector's optical axis. The second camera was positioned at the same angle to the projector's optical axis. The resolution of the cameras was 2 Mpix. The resolution of the projector was 1280 × 720p (HD-Ready). The system was arranged so as to enable the measurement of objects within a 180 × 180 × 100 mm sample volume. The projector emitted white light. Color cameras were used. A single scan produced a point cloud, with the position of each point in space given in mm. The units were chosen at the stage of entering parameters in the system, before calibration. A single scan comprised data calculated based on information from the two cameras. The double-camera setting enabled the simultaneous measurement of the object from two directions, minimizing the unscanned portion of the object. The axial accuracy of the measurement was 0.1 mm, and the mean distance between measurement points in the measurement plane was also 0.1 mm. The system was calibrated so that both scanners (comprising the separate cameras and the common projector) worked in the same coordinate system. Additionally, as part of the calibration, one of the axes was set so as to pass through the rotation axis of the rotary table, and the remaining two axes were parallel to the surface of the table. This enabled the automatic merging of the data. The slight deviations in the above parameters were adjusted for algorithmically. After each measurement, the table rotated by 15°; therefore, 24 subsequent measurements provided a 360° recording of the object's shape. After 24 measurements, the table rotated by another 15° to return to its initial position. In the measurement process, apart from the dimensions of the objects, information on the objects' texture was also recorded. A complete revolution of the table comprised 14.400 microrotations, which allowed positioning in 0.025° steps.

A very significant factor in the measurements was finding the appropriate amplification and shutter settings, so as to enable the scanning of the largest surface possible. The need for entering certain parameters was due to the various colors of the bones measured. Therefore, values were adjusted experimentally for each object. All data processing and the scanning process itself were performed using the FRAMES software, developed by a team working at the Warsaw University of Technology Division of Virtual Reality.

## 3. Results

**Figure 2:**
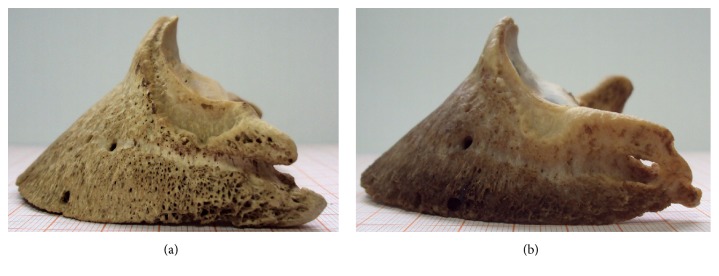
The macerated coffin bones: (a) well-macerated coffin bone with fat completely removed; (b) coffin bone covered by a fat layer, reflecting light.

**Figure 3:**
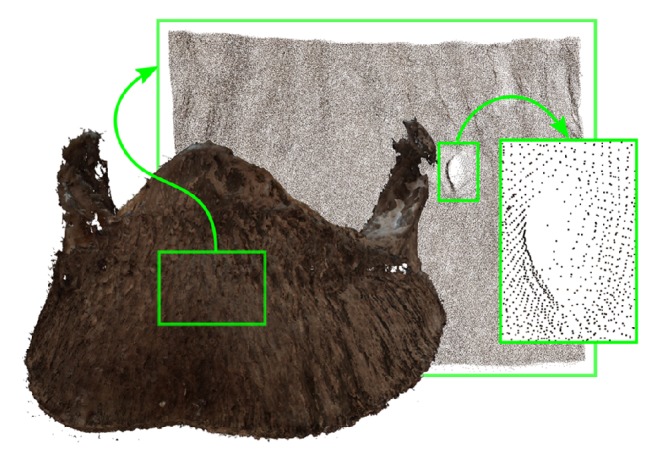
Example measurement results after preprocessing and merging of the directional point clouds.

During the measurements, it turned out that the three-dimensional scanning method used is better suited for measuring light bones, whose surfaces do not significantly reflect or absorb light. Therefore, better scans were obtained from well-macerated bones, with fat completely removed (Figure 2(a)). Darker, more reflective bones, with a layer of fat, were more difficult to scan (Figure 2(b)). Thus, it was found that any fat layer should be removed from the scanned bone surfaces; that is, the pretreatment of bones is extremely important. Furthermore, after treatment, the surfaces of some bones were found to be porous (Figure 2(a)). The optical system was capable of scanning and measuring the pores only to the depth where the light from the projector was still observable by the cameras. It was found that the depth of pore measurement could be increased by reducing the distance between the cameras and the projector; however, the smaller distance resulted in reduced axial accuracy.  Figure 3 shows an example measurement result after initial processing. It also shows subsequent enlargements of selected areas.

**Figure 4:**
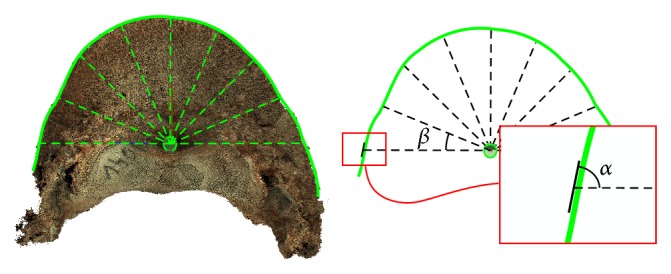
Method for determining angle values for the plantar margin of the coffin bone.

After the measurement of a bone, the points were projected onto a plane, corresponding to the surface of the rotary table. Thus, a two-dimensional image was produced. At the next stage, the bone image was rotated so as to achieve approximate horizontal symmetry. The image of the right coffin bone was flipped horizontally. Subsequently, the images of the left and right coffin bones from the same animal were superimposed using graphics software. The right coffin bone image was shifted and/or rotated so as to achieve the best overlay of the two images. For each image, we delineated the plantar margin of the bone and determined its middle point, which then constituted the zero point of a coordinate system. The margin line was transformed into polar coordinates. In the ±90° angle range, 9 measurement points were defined, spaced 22.5° apart (Figure 4). At each point, the straight line best fitting the given fragment of the margin was calculated. The fragment comprised points located within ±22.5° from a measurement point. The angle range for the bone fragment was chosen experimentally and as wide as possible, so as to minimize the influence of local distortions of the line, resulting, for example, from minor damage to the bone occurring, for example, during the removal of soft tissue.

**Figure 5:**
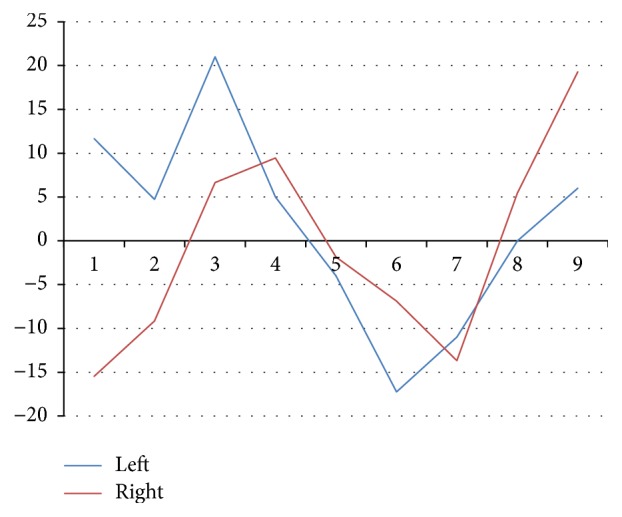
Angle values at the measurement points for the plantar margins of the left and right coffin bones from one horse in the study.

With the equation of the best-fit line known, the angle between this line and the line passing through the current measurement point and the center of gravity was calculated. Thus, angle values for all measurement points for the left and right coffin bones were obtained. Example results are shown in Figure 5. On this basis, the difference between angles in each measurement point and the mean difference from all points were calculated for each pair of bilateral bones. The results are shown in Table 1.

Pearson's linear correlation coefficient was calculated for correlations between mean angle differences and the horses' age and weight. The coefficients were 0.24 (*p* = 0.15) and 0.14 (*p* = 0.41), respectively. The above results indicate a weak correlation between age and bilateral third-phalanx asymmetry in terms of the angle range of the plantar margins of coffin bones in the studied cold-blood horse population and no correlation between body weight and the asymmetry described.

## 4. Discussion

The results of studies on asymmetry between bilateral bones of the equine autopodium are equivocal. Analyses were performed both on live animals [25–27] and on isolated bones [28–30]. Studies on live horses were based on caliper measurements of corresponding sections of bilateral limbs, with particular anatomical structures of the measured bones constituting reference points. The studies were performed on race horses and ponies and investigated the presence of any limb asymmetry resulting from uneven load distribution between the limbs during training. Results indicated that bilateral limb asymmetry in horses was unrelated to the type of training and that it might be typical in the species regardless of breed and use. The third metacarpal and third metatarsal bones were longer on the right side, while proximal phalanges were wider on the left side [27]. Other researchers assessed the cortical thickness of the third metacarpal bone in race horses. The measurements were performed on appropriate radiographs and showed that cortical thickness was higher on the right side [25]. Dymock and Pauwels (2012) emphasized the need for studies on the bilateral asymmetry of equine limbs, due to the equivocality of data [28]. Another study, on proximal phalanges in the thoracic limb, showed no significant differences in BMD or BMC between right and left limbs [29].

Available literature lacks reports on asymmetry between bilateral coffin bones in horses. The present study is the first one on the subject. Previous studies only assessed bilateral asymmetry between hooves [27, 31, 32], which was shown to be strictly correlated with asymmetries between particular limb segments [32].

The choice of an appropriate equine population was an important aspect of the present study. The animals selected had not undergone any training that could contribute to unequal strain on the limbs, as is the case, for example, in race horses. In the study, no correlation was found between body weight and bilateral coffin bone asymmetry in terms of the angle range of the plantar margin. Remarkably, at the same time, a correlation was shown between bilateral coffin bone asymmetry and age. This observation seems to confirm the hypothesis put forward by Leśniak, stating that asymmetry in the structure of bilateral equine limbs and in the load placed on each side occurs regardless of the type of training [27, 33]. Simultaneously, analyses by other authors indicate that unequal strain on the limbs due, for example, to lameness does contribute to asymmetry between hooves [34, 35]. The three-dimensional scanning technique can also be used for measuring the width and depth of any openings in the bone tissue. For this usage, the distances between the cameras and the projector should be reduced.

Our observations also showed that the initial treatment of bones is a very important stage of the process, as the surface should be as fat-free and nonreflective as possible. The equine autopodia for traditional osteometric measurement may be macerated for a shorter time [16, 19]. However, coffin bones for three-dimensional scanning should be macerated for 48 hours, in a temperature of 95–99°C. This treatment time is sufficient, but, during the treatment, fat should be regularly removed from water surface, and, after its completion, the bones should be rinsed thoroughly to remove any fat residue.

## 5. Conclusions

Three-dimensional scanning is a new technique that may be a valuable complement to traditional measurement methods. The present study was a pilot analysis of bilateral third phalanges of equine thoracic limbs. As it turned out, three-dimensional scanning can be used to effectively determine the angle range for a selected portion of the studied bone. This use of the method is of particular value in osteometry, as it enables the performance of additional measurements that cannot be easily performed using traditional measurement techniques. Therefore, it offers broader possibilities for bone structure analysis and angle distribution assessment and, thus, for detailed analysis of bilateral bone asymmetry, but the initial treatment of bones is a very important stage of the process. The surface should be as fat-free and nonreflective as possible. Our researches indicate a weak correlation between age and bilateral third-phalanx asymmetry in terms of the angle range of the plantar margins of coffin bones in the studied cold-blood horse population and no correlation between body weight and the asymmetry described.

The presence of bilateral third-phalanx asymmetry in horses has not yet been fully explained; further studies are needed, involving a more general population and including measurements made possible by the use of three-dimensional scanning.

## Figures and Tables

**Table 1:** Mean differences between angles for the right and left coffin bones from each animal in the studied cold-blood horse population.

Number	Age [years]	Body weight [kg]	Mean difference between angles for the right and left coffin bones
(1)	1	491	1.91
(2)	1	346	1.84
(3)	2	543	2.88
(4)	2	559	1.70
(5)	3	424	1.22
(6)	5	523	0.95
(7)	6	362	0.81
(8)	6	505	2.08
(9)	7	586	1.25
(10)	7	793	1.90
(11)	8	479	1.13
(12)	8	748	1.93
(13)	10	602	1.45
(14)	10	680	1.23
(15)	10	651	1.15
(16)	10	542	2.70
(17)	11	515	2.33
(18)	11	646	1.87
(19)	12	549	1.37
(20)	12	737	1.99
(21)	12	652	2.39
(22)	12	655	1.51
(23)	13	549	1.93
(24)	13	679	2.04
(25)	14	865	2.23
(26)	14	568	1.91
(27)	17	608	2.23
(28)	17	666	3.31
(29)	18	366	3.18
(30)	18	567	2.11
(31)	18	584	1.52
(32)	18	658	1.03
(33)	19	646	2.07
(34)	20	523	1.44
(35)	23	543	1.34
(36)	23	648	1.99
(37)	25	484	3.87
(38)	25	461	0.94

## References

[1] Chen S. Y., Li Y. F., Zhang J. (2008). Vision processing for realtime 3-D data acquisition based on coded structured light. *IEEE Transactions on Image Processing*.

[2] Ramsey G. (2011). *Equine hoof biomechanics [M.S. dissertation]*.

[3] Yang G., Sun C., Wang P., Xu Y. (2014). High-speed scanning stroboscopic fringe-pattern projection technology for three-dimensional shape precision measurement. *Applied Optics*.

[4] Takasaki H. (1970). Moire topography. *Applied Optics*.

[5] Posdamer J. L., Altschuler M. D. (1982). Surface measurement by space-encoded projected beam systems. *Computer Graphics and Image Processing*.

[6] Brenner C., Boehm J., Guehring J. Photogrammetric calibration and accuracy evaluation of a cross-pattern stripe projector.

[7] Jason Geng Z. (1996). Rainbow three-dimensional camera: new concept of high-speed three-dimensional vision systems. *Optical Engineering*.

[8] Geng J. Method and apparatus for 3D imaging using light pattern having multiple sub-patterns.

[9] Zhang L., Curless B., Seitz S. Rapid shape acquisition using color structured light and multi-pass dynamic programming.

[10] Petriu E. M., Sakr Z., Spoelder H. J., Moica A. Object recognition using pseudo-random color encoded structured light.

[11] Sitnik R., Kujawińska M., Woźnicki J. (2002). Digital fringe projection system for large-volume 360-deg shape measurement. *Optical Engineering*.

[12] Gabriel A., Jolly S., Detilleux J., Dessy-Doize C., Collin B., Reginster J.-Y. (1998). Morphometric study of the equine navicular bone: variations with breeds and types of horse and influence of exercise. *Journal of Anatomy*.

[13] Cumming D. H. M., Cumming G. S. (2003). Ungulate community structure and ecological processes: body size, hoof area and trampling in African savannas. *Oecologia*.

[14] Komosa M., Moliński K., Godynicki S. (2006). The variability of cranial morphology in modern horses. *Zoological Science*.

[15] Komosa M., Purzyc H. (2009). Konik and Hucul horses: a comparative study of exterior measurements. *Journal of Animal Science*.

[16] Abdunnabi A. H., Ahmed Y. A., Philip C. J., Davies H. M. S. (2012). Morphometrical variations of the carpal bones in thoroughbreds and ponies. *Journal of Veterinary Medicine Series C: Anatomia Histologia Embryologia*.

[17] Komosa M., Purzyc H., Fra̧ckowiak H. (2013). Changes in navicular bone (os sesamoideum distale) shape in horses as a result of pathological alterations. *Folia Biologica (Poland)*.

[18] Dzierzęcka M., Komosa M. (2013). Variability of the proximal phalanx in warmblood and coldblood horses—morphological and structural analyses. *Belgian Journal of Zoology*.

[19] Alrtib A. M., Philip C. J., Abdunnabi A. H., Davies H. M. S. (2013). Morphometrical study of bony elements of the forelimb fetlock joints in horses. *Journal of Veterinary Medicine Series C: Anatomia Histologia Embryologia*.

[20] Auerbach B. M., Ruff C. B. (2006). Limb bone bilateral asymmetry: variability and commonality among modern humans. *Journal of Human Evolution*.

[21] Leamy L. J., Meagher S., Taylor S., Carroll L., Potts W. K. (2001). Size and fluctuating asymmetry of morphometric characters in mice: their associations with inbreeding and t-haplotype. *Evolution*.

[22] John L., Yin Y. Y., Chow David Y. W., Kwong C. L. (2012). Planar scintigraphy in assessment of mandibular asymmetry: unilateral condylar hyperplasia vs asymmetric mandibular hyperplasia. *Journal of Biomedical Science and Engineering*.

[23] Dzierzęcka M., Purzyc H., Charuta A. (2016). Evaluation of distal phalanx formation and association with front hoof conformation in coldblooded horses. *Biologia*.

[24] Parliament of the Republic of Poland: The Act of 21 August 1997 on the protection of animals. Dz. U. 1997 Nr 111 poz. 724 with modifications 2012, http://isap.sejm.gov.pl/DetailsServlet?id=WDU19971110724

[25] Davies H. M. S., Watson K. M. (2005). Third metacarpal bone laterality asymmetry and midshaft dimensions in Thoroughbred racehorses. *Australian Veterinary Journal*.

[26] Tóth P., Horváth C., Ferencz V. (2010). Assessment of the mineral density and mineral content of the equine third metacarpal and first phalanx bone by dual energy x-ray absorptiometry. *Acta Veterinaria Hungarica*.

[27] Leśniak K. (2013). Directional asymmetry of facial and limb traits in horses and ponies. *Veterinary Journal*.

[28] Dymock D. C., Pauwels F. E. T. (2012). Investigation into the morphology of the third metacarpal bone in the horse. *New Zealand Veterinary Journal*.

[29] Dzierzęcka M., Charuta A. (2012). The analysis of densitometric and geometric parameters of bilateral proximal phalanges in horses with the use of peripheral quantitative computed tompgraphy. *Acta Veterinaria Scandinavica*.

[30] Dzierzecka M., Charuta A. (2012). Comparison of the proximal phalanx parameters in warmblood and coldblood horses with the use of peripheral quantitative computed tomography. *Bulletin of the Veterinary Institute in Pulawy*.

[31] Roland E., Stover S. M., Hull M., Dorsch K. (2003). Geometric symmetry of the solar surface of hooves of thoroughbred racehorses. *American Journal of Veterinary Research*.

[32] Wilson G. H., McDonald K., O'Connell M. J. (2009). Skeletal forelimb measurements and hoof spread in relation to asymmetry in the bilateral forelimb of horses. *Equine Veterinary Journal*.

[33] Heaps L. A., Franklin S. H., Colborne G. R. (2011). Horizontal moment around the hoof centre of pressure during walking on right and left circles. *Equine Veterinary Journal*.

[34] Oosterlinck M., Gasthuys F., Back W., Pille F. (2013). Does long-term unilateral circling affect locomotor symmetry in ponies used for carousel rides*α*. *Veterinary Journal*.

[35] Wilson A., Agass R., Vaux S. (2016). Foot placement of the equine forelimb: relationship between foot conformation, foot placement and movement asymmetry. *Equine Veterinary Journal*.

